# Genetic Profiling of MC3T3-E1 Cells in Different Media: Implications for In Vitro Screening Development

**DOI:** 10.3390/biomedicines13020489

**Published:** 2025-02-17

**Authors:** Makoto Izumiya, Hidehiko Nobuoka, Hono Endo, Rintaro Ueno, Masaki Mimura, Naoto Saito, Hisao Haniu

**Affiliations:** 1 Institute for Biomedical Sciences, Interdisciplinary Cluster for Cutting Edge Research, Shinshu University, 3-1-1 Asahi, Matsumoto 390-8621, Nagano, Japan; 21hb401k@shinshu-u.ac.jp (M.I.); 23bs217f@shinshu-u.ac.jp (H.N.); 24bs206d@shinshu-u.ac.jp (H.E.); 23bs204d@shinshu-u.ac.jp (R.U.); 23bs223a@shinshu-u.ac.jp (M.M.); saitoko@shinshu-u.ac.jp (N.S.); 2 Biomedical Engineering Division, Graduate School of Medicine, Science and Technology, Shinshu University, 3-1-1 Asahi, Matsumoto 390-8621, Nagano, Japan; 3 Biomedical Engineering Division, Graduate School of Science and Technology, Shinshu University, 3-1-1 Asahi, Matsumoto 390-8621, Nagano, Japan

**Keywords:** in vitro, biomaterial, culture medium, bone formation, calcification, MC3T3-E1, primary osteoblasts, screening methods

## Abstract

**Background/Objectives**: The translation of in vitro biomaterial evaluations into successful clinical applications often fails due to discrepancies with in vivo results. Previously, we demonstrated that differences in culture medium conditions influence the bone formation process. This study aimed to investigate the influence of culture media on gene expression during calcification induction in osteoblasts. **Methods**: Using MC3T3-E1 cells cultured in α Minimum Essential Medium without L-ascorbic acid (αMEM(−)) and Dulbecco’s Modified Eagle Medium (DMEM), we screened gene expression profiles through microarray analysis and validated key findings with quantitative PCR. Additionally, we compared these gene expression patterns with those in primary osteoblasts (POBs) cultured under the same medium conditions. **Results**: The results revealed distinct gene expression profiles in MC3T3-E1 cells depending on the culture medium, while POBs exhibited minimal differences between media, except for the gene *Alpl*. In αMEM(−), *Alpl* expression in POBs was significantly increased approximately 4-fold via calcification stimulation (*p* < 0.0001). POBs cultured in DMEM showed calcification appearance differing from the αMEM(−) condition, even though no significant increase in *Alpl* expression via calcification stimulation was observed. **Conclusions**: Differences in media appear to remarkably impact osteoblast gene expression and mineralization. These findings may help improve biomaterial evaluation when transitioning from in vitro assessments to in vivo evaluations. Moreover, our results suggest the possibility that gene expression differences observed in MC3T3-E1 cells reflect the diverse bone formation processes in vivo. Focusing on these genes could facilitate the development of screening methods for bone formation, supporting future clinical applications in orthopedics.

## 1. Introduction

The development of new biomaterials and tissue engineering treatments for bone-related diseases relies heavily on animal experiments to evaluate safety and efficacy. However, there is an increasing need to minimize animal use while conducting research efficiently, as mandated by modern scientific standards [1]. To address this, it is essential to establish in vitro evaluation methods that can accurately replicate and screen the diverse bone formation processes occurring in the body.

Since the establishment of culture technology for bone-forming cells [2], functional evaluations have been widely used to assess the biological effectiveness of implant materials on osteoblasts. These evaluations focus on processes such as osteoblast differentiation and bone mineralization and have become the cornerstones of in vitro testing. Despite this, very few new implant materials have been successfully translated into clinical applications based solely on positive in vitro results, such as enhanced osteoblast function. This disconnect is primarily attributed to the frequent discrepancies between in vitro and in vivo functional evaluations [3,4,5], which is thought to be due in part to the complex physiological environment within the body. Currently, no consensus exists on an in vitro bone formation model capable of accurately reproducing the complex physiological environment of bone formation in the body [6]. This remains a significant challenge for researchers and developers.

The MC3T3-E1 preosteoblast cell line is among the most widely used models for studying in vitro bone formation. It has been cited in over 4000 publications, including studies involving its derivatives, highlighting its popularity in the field of bone research [7]. However, MC3T3-E1 cells are known for their instability [8,9]. Wang et al. established a subclone of MC3T3-E1 cells using α Minimum Essential Medium (αMEM) without L-ascorbic acid (αMEM(−)) [9], which differs slightly from the original αMEM formulation used during cell line establishment. This adjustment was based on findings that L-ascorbic acid supports collagen matrix synthesis and stimulates the expression of osteoblast differentiation markers. Despite these considerations, medium conditions are often overlooked in in vitro evaluations of biomaterials [10,11], resulting in significant variability across studies.

Our previous study demonstrated that the freshness and composition of the culture medium significantly influenced osteoblast differentiation-related gene expression, cell proliferation, alkaline phosphatase activity, and mineralization in MC3T3-E1 cells [12]. Specifically, we observed that commercially available liquid αMEM contained inactive L-ascorbic acid, showing no significant differences compared to αMEM(−). However, bone formation processes and mineralized nodule formation induced by MC3T3-E1 cells varied depending on the type of medium used. These findings suggest that multiple in vitro bone formation processes may exist. In contrast, bone formation in vivo is equally diverse, involving distinct pathways such as membranous and endochondral ossification. Additionally, bone formation occurs under various physiological and pathological conditions, including development, remodeling for homeostasis, fracture healing, and ectopic ossification [13,14]. It remains unclear whether in vitro bone formation under different medium conditions mirrors any specific in vivo processes or corresponds to particular states of in vivo bone formation.

Given these challenges, there is an urgent need to understand how culture medium conditions influence in vitro bone formation processes and their relevance to in vivo mechanisms. This study aims to identify gene expression differences in MC3T3-E1 cells under varying culture conditions and to assess their correspondence to primary osteoblast (POB) behavior, with the goal of improving in vitro models for bone formation research.

## 2. Materials and Methods

### 2.1. Cells

#### 2.1.1. MC3T3-E1

MC3T3-E1 (cell no. RCB1126) cells were obtained from RIKEN BRC (Ibaraki, Japan). The cells were expanded for two passages in αMEM (Thermo Fisher Scientific, Waltham, MA, USA) supplemented with 10% fetal bovine serum (FBS) (Thermo Fisher Scientific, Waltham, MA, USA), following the recommended culture conditions. The expanded cells were then aliquoted into small portions and stored at −85 °C.

#### 2.1.2. MC3T3-E1 Clone

MC3T3-E1 clone cells were generated from the parental MC3T3-E1 cells using the limiting dilution method. Briefly, the cell concentration of MC3T3-E1 cells was measured using a Trypan Blue solution (Sigma-Aldrich, Saint Louis, MO, USA) with a TC20^TM^ automated cell counter (BIO-RAD, Hercules, CA, USA). In accordance with the published literature [15], the cells were diluted with αMEM (Nacalai Tesque, Kyoto, Japan) supplemented with 10% FBS to 2 cells/mL, and then seeded in 100 µL portions onto 96-well plates (Sarstedt, Numbrecht, Germany). Once single colonies had formed, the cells were expanded, aliquoted into small portions, and stored at −85 °C.

#### 2.1.3. Primary Osteoblasts

**Figure 1:**
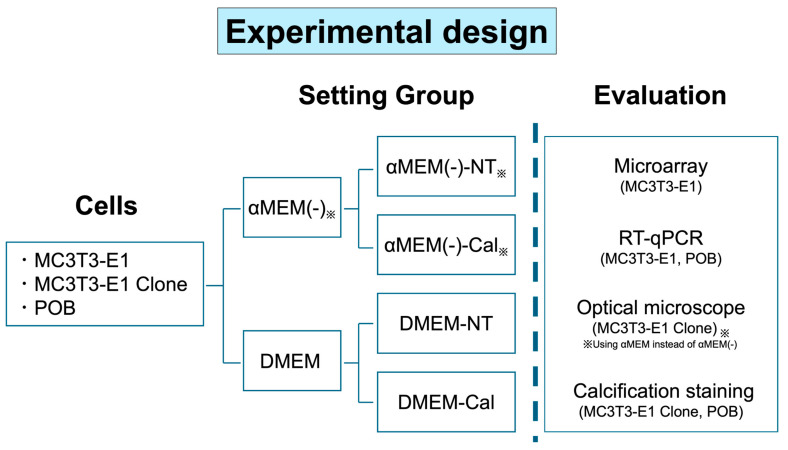
Simple visualization of the experimental design. Immediately after thawing or harvesting, each cell type was divided into two medium conditions: (1) α Minimum Essential Medium (αMEM) or αMEM without L-ascorbic acid (αMEM(−)); and (2) Dulbecco’s Modified Eagle Medium (DMEM). Then, each medium was further divided into two medium conditions: non-treated media (NT) and media supplemented with L-ascorbic acid and β-glycerophosphate to stimulate calcification (Cal) for each evaluation. Primary osteoblast: POB. The symbol in the figure indicates optical microscope evaluation used αMEM instead of αMEM(−).

Based on the national and institutional regulations and guidelines, all animal experiment procedures were reviewed by the Committee for Animal Experiments and approved by the president of Shinshu University (Approval Number 021078). Primary osteoblasts (POBs) were isolated from the calvaria of C57BL6/N neonatal mouse pups within 1 week of birth, following a modified protocol based on Bakker and Klein-Nulend [16]. The collected calvarial bones were washed, fragmented into 1–2 mm pieces, and digested using a solution containing 0.05 *w*/*v*% Trypsin-0.53 mmol/L EDTA 4Na solution (FUJIFILM Wako, Osaka, Japan), and 0.064% collagenase II (Worthington Biochemical, Lakewood, NJ, USA). The reaction was performed at 37 °C for 100 min with intermittent stirring every 10 min. The reaction was halted using Dulbecco’s Phosphate-Buffered Saline (DPBS) containing FBS, and the cell suspension was filtered through a 100 μm cell strainer (Greiner Bio-One, Kremsmuster, Austria). The filtrate was centrifuged at 300× *g* for 5 min, and the resulting pellet was resuspended in DPBS. The cell suspension was divided equally and cultured in two different media as recommended in the POB harvesting protocol [16,17]: αMEM(−) (Thermo Fisher Scientific, Waltham, MA, USA) supplemented with 10% FBS and Dulbecco’s Modified Eagle Medium (DMEM) (FUJIFILM Wako, Osaka, Japan). The following day, the medium was replaced with fresh media. Cells were passaged upon reaching 80~90% confluency, and cells within two passages were used for experiments. The experimental design is shown in Figure 1.

### 2.2. DNA Microarray

MC3T3-E1 cells were thawed and cultured in two different media–αMEM(−) and DMEM supplemented with 10% FBS–and then seeded onto 12-well plates (Sarstedt, Numbrecht, Germany) at a density of 6.0 × 10^4^ cells/mL (*n* = 4). The following day, the cells were divided into two treatment groups: (1) non-treated medium for growth; and (2) calcification-inducing medium, which contained 100 μg/mL of L-ascorbic acid (Nacalai Tesque, Kyoto, Japan) and 5 mM of β-glycerophosphate (Calbiochem, La Jolla, CA, USA). 3 days after induction, total RNA was extracted using a RNA extraction kit (Nippon Genetics, Tokyo, Japan) according to the manufacturer’s protocol. Equal amounts of RNA (5 µL per sample) from four replicates were pooled for each group. DNA microarray analysis was conducted by Macrogen Inc. (Seoul, Republic of Korea) using the Clariom™ S assay for mice (Thermo Fisher Scientific Affymetrix, Santa Clara, CA, USA). In brief, the Affymetrix whole-transcript expression array process was performed according to the manufacturer’s protocol (GeneChip Whole Transcript PLUS Reagent Kit (Thermo Fisher Scientific Affymetrix, Santa Clara, CA, USA)). cDNA was synthesized using the GeneChip WT (whole-transcript) amplification kit according to the manufacturer’s instructions. Sense cDNA was fragmented and biotinylated with TdT (terminal deoxynucleotidyl transferase) using the GeneChip WT terminal labeling kit. Approximately 5.5 μg of labeled DNA target was hybridized to the Affymetrix GeneChip array for 16 h at 45°C. The hybridized array was washed and stained in the GeneChip Fluidics Station 450 and scanned with a GCS3000 scanner (Thermo Fisher Scientific Affymetrix, Santa Clara, CA, USA). The calculation of probe cell intensity data and the generation of analysis files were performed using the Affymetrix^®^ GeneChip Command Console^®^ software ver. 4.0 (Thermo Fisher Scientific Affymetrix, Santa Clara, CA, USA). The analysis files were normalized and analyzed using Analysis Power Tools ver. 2.11.6 (Thermo Fisher Scientific Affymetrix, Santa Clara, CA, USA). After comparing the processed data of each group, the top 10 genes with increased expression on day 3 after calcification-inducing treatment were identified for each medium.

### 2.3. RT-Quantitative PCR

MC3T3-E1 cells and POBs were cultured in two different media–αMEM(−) and DMEM supplemented with 10% FBS–immediately after thawing or harvesting, respectively. The cells were seeded onto 12-well plates at a density of 6.0 × 10^4^ cells/mL and 8.0 × 10^4^ cells/mL, respectively, and divided into two groups for each medium: (1) non-treated medium (MC3T3-E1: *n* = 6, POB: *n* = 12); and (2) calcification-inducing medium (MC3T3-E1: *n* = 6, POB: *n* = 12). 3 days post-induction, total RNA was extracted using an RNA extraction kit, followed by cDNA synthesis using ReverTra Ace qPCR RT Master Mix with gDNA Remover (Toyobo, Osaka, Japan). Gene expression analysis was performed using THUNDERBIRD Next SYBR qPCR Mix (Toyobo, Osaka, Japan) on a Step One Plus Real-Time PCR System (Thermo Fisher Scientific, Waltham, MA, USA). The primers for gene expression analysis (purchased from Takara Bio Inc. (Shiga, Japan)) are listed in Table 1. Glyceraldehyde-3-phosphate dehydrogenase (*Gapdh*) was used as the housekeeping gene, and relative gene expression levels were quantified using the ΔΔCt method.

### 2.4. Optical Microscopy

MC3T3-E1 clone cells were seeded at a density of 6.0 × 10^4^ cells/mL in two different media, αMEM and DMEM, immediately after thawing. After 1 day, the cells were divided into two groups for each medium: non-treated medium and calcification-inducing medium. Once the calcification induction process began, the medium was replaced every 3 days. On day 21 of calcification induction, the cellular states under each medium condition were observed using a Leica DM IL LED inverted phase contrast microscope (Leica Microsystems, Wetzlar, Germany).

### 2.5. Calcification Evaluation

MC3T3-E1 clone cells were cultured in αMEM(−) and DMEM and seeded onto 48-well plates (Sarstedt, Numbrecht, Germany) at a density of 1.0 × 10^5^ cells/mL (*n* = 6). POB cells, cultured in αMEM(−) and DMEM immediately after collection, were seeded onto collagen-coated 48-well plates (IWAKI, Shizuoka, Japan) at a density of 8.0 × 10^4^ cells/mL (*n* = 12). After 1 day of culture, the medium for both cell types was divided into two groups: non-treated medium and calcification-inducing medium. To account for cell detachment, half of the medium was replaced every 3 days during the calcification process. Calcification in MC3T3-E1 clone cells was evaluated 21 days after induction, while calcification in POBs was assessed using Alizarin Red S staining after 35 days. The evaluation procedure involved washing cells with DPBS, followed by fixation with ice-cold methanol for 20 min. After removing the methanol, cells were washed with pure water and stained with a 1% Alizarin Red S (Sigma-Aldrich, Saint Louis, MO, USA) solution (pH 6.4) for 5 min. Excess stain was removed by washing with pure water, and the plate was left to dry. For quantification, calcified nodules were dissolved in 5% formic acid (FUJIFILM Wako, Osaka, Japan) with agitation for 10 min. The absorbance of the resulting eluate was measured at 405 nm to determine calcification levels with a PlateReader AF2200 (Eppendorf, Hamburg, Germany).

### 2.6. Statistical Analysis

Statistical analysis was performed using GraphPad Prism Ver. 7.02 (Graph Pad Software Inc., San Diego, CA, USA). The Shapiro–Wilk test was performed to test for normality. Welch’s *t*-test was applied for comparisons between two groups when normality was established, while the Mann–Whitney U test was used for non-normal data. A 95% confidence interval was assumed, with the significance level set at *p* < 0.05.

## 3. Results

### 3.1. Comprehensive Gene Analysis of MC3T3-E1 Cells Cultured in Different Media After Calcification Stimulation

MC3T3-E1 cells were cultured in αMEM(−) and DMEM, respectively, and their gene expression profiles were compared 3 days after calcification-inducing stimulation using microarray analysis. The results revealed that the top upregulated genes differed markedly between the two culture media. In the αMEM(−) culture, the most upregulated genes, in order, were *Lum*, *Mmp13*, *Ibsp*, *F13a1*, *Alpl*, *Pmel*, *Slc27a1*, *Crabp1*, *Mmp9*, and *Gm11099* (a predicted gene) (Table 2). Notably, none of these genes exhibited more than a two-fold upregulation in the DMEM culture.

Conversely, the most upregulated genes in the DMEM culture were *Dusp4*, *Dmp1*, *Lrp8*, *Adgrl3*, *Mnda*, *Olfr775*, *Prkg2*, *Fam64a*, *Gm13212* (a predicted gene), and *Gpnmb*, in that order. Among these, only *Dmp1* and *Gpnmb* showed approximately a two-fold increase in the αMEM(−) culture, while the other genes did not exceed a two-fold upregulation.

### 3.2. Validation of Microarray Data Using RT-qPCR

**Figure 2:**
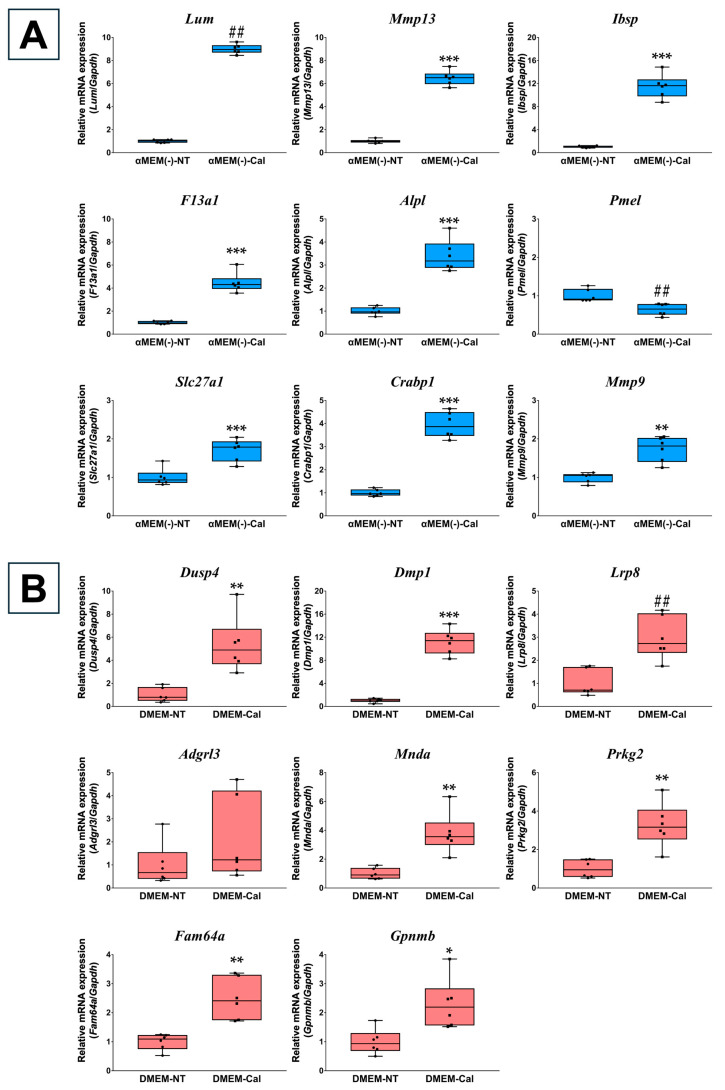
RT-qPCR results for top genes in MC3T3-E1 cells cultured in different media. (**A**) RT-qPCR was performed on MC3T3-E1 cells cultured in α Minimum Essential Medium without L-ascorbic acid (αMEM(−)) to validate the top genes identified under αMEM(−) culture conditions from the microarray results. Gene expression was normalized to *Gapdh* and calculated relative to the αMEM(−) non-treated group (αMEM(−)-NT). (**B**) RT-qPCR was performed on MC3T3-E1 cells cultured in Dulbecco’s Modified Eagle Medium (DMEM) to validate the top genes identified under DMEM culture conditions from the microarray results. Gene expression was normalized to *Gapdh* and calculated relative to the DMEM non-treated group (DMEM-NT). Data are presented as box plots showing median values, with whiskers representing the minimum and maximum values, and all individual points (*n* = 6) are shown. Statistical significance was determined using the Welch’s *t*-test for normally distributed data (* *p* < 0.05, ** *p* < 0.01, *** *p* < 0.001) and the Mann–Whitney U test for non-normal distributions (## *p* < 0.01).

The microarray results were validated using RT-qPCR for the top upregulated genes, excluding predicted genes, in each medium after 3 days of calcification-inducing stimulation. In MC3T3-E1 cells cultured in αMEM(−), all selected top genes, except *Pmel,* were significantly upregulated. For *Slc27a1* and *Mmp9*, expression levels were upregulated by 1.71-fold (*p* = 0.0008) and 1.73-fold (*p* = 0.0016), respectively, which, while less than two-fold, aligned closely with the microarray results (Figure 2A). In MC3T3-E1 cells cultured in DMEM, all selected top genes, except *Adgrl3* and *Olfr775*, were significantly upregulated. *Olfr775* was undetected (cutoff Ct value > 40). These findings were consistent with the microarray results (Figure 2B).

### 3.3. Optical Microscopy of MC3T3-E1 Clone Cells Cultured in Different Media

**Figure 3:**
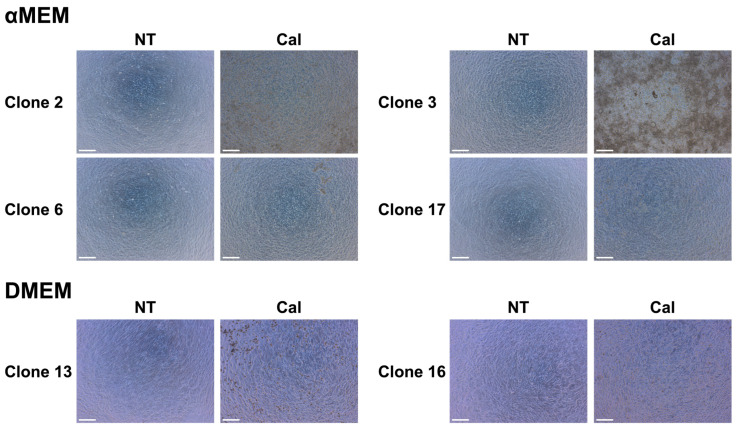
Optical microscopy of MC3T3-E1 clone cells 21 days after calcification induction in each medium. MC3T3-E1 clone cells cultured in α Minimum Essential Medium (αMEM) and Dulbecco’s Modified Eagle Medium (DMEM) were observed using optical microscopy 21 days after calcification induction to compare their states. The medium was replaced every 3 days during the induction period. Representative examples of different appearances are shown for Clones 2, 3, 6, 13, 16, and 17. Scale bar: 200 µm. Non-treated group: NT; calcification induction group: Cal.

Seventeen types of MC3T3-E1 clone cells were cultured in αMEM and DMEM, and their morphological characteristics were observed under an optical microscope 21 days after calcification induction. Significant differences were observed between clones and medium conditions (Figure 3). In the αMEM-calcification induction (Cal) group, Clone 2 exhibited no island-like nodule formation, and brown regions covered a wide area. Clone 3 displayed large black and brown island-like nodules across a wide area, whereas Clone 6 showed black island-like nodules only in some regions. The αMEM-Cal group of Clone 17 was similar to that of Clone 2 but had fewer brown areas overall. In the DMEM-Cal group, Clone 13 showed dark black spherical particles distributed over a wide area, whereas Clone 16 exhibited yellowish-brown spherical particles that were more prevalent and covered a larger area than in Clone 13.

### 3.4. Calcification of MC3T3-E1 Clone Cells in Different Media

**Figure 4:**
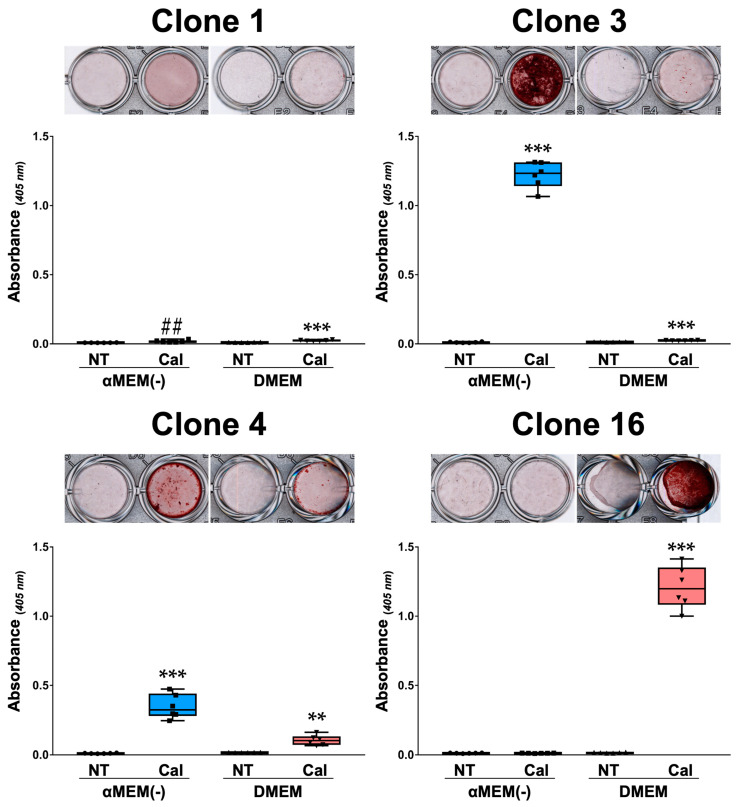
Calcification evaluation of MC3T3-E1 clone cells 21 days after calcification induction in each medium. MC3T3-E1 clone cells cultured in α Minimum Essential Medium without L-ascorbic acid (αMEM(−)) and Dulbecco’s Modified Eagle Medium (DMEM) were stained with Alizarin Red S 21 days after calcification induction to compare their calcification response. After induction, half of the medium was replaced every 3 days. Representative images of Alizarin Red S-stained wells under each medium condition are shown, along with a graph of the absorbance at 405 nm, measured from the eluted mineral nodules using 5% formic acid, stirred for 10 min. Data are presented as box plots showing the median, with whiskers representing the minimum and maximum values, and all individual points (*n* = 6) are shown. Statistical significance was evaluated using Welch’s *t*-test for normally distributed data (** *p* < 0.01, *** *p* < 0.001) and the Mann–Whitney U test for non-normal distributions (## *p* < 0.01). Non-treated group: NT, calcification induction group: Cal.

MC3T3-E1 clone cells cultured in αMEM(−) and DMEM were stained with Alizarin Red S on the 21st day after calcification-inducing stimulation to compare the calcification reaction. The calcification response varied by clone and medium type. Representative calcification images of MC3T3-E1 clone cells with notable differences between media are shown alongside the quantification of calcification dissolved in formic acid (Figure 4). In Clone 1, slight staining was observed in both αMEM(−) and DMEM Cal groups compared to their respective non-treated (NT) groups, but no visible calcified nodules were detected. Clone 3 exhibited visible calcified nodules in the αMEM(−)-Cal group (*p* < 0.0001), with a slightly but significantly higher calcification level in the DMEM-Cal group than in the D-NT group (*p* < 0.0001). In contrast, in Clone 16, visible calcified nodules were observed in the DMEM-Cal group (*p* < 0.0001), whereas no significant difference was detected between the αMEM(−)-Cal and αMEM(−)-NT groups (*p* = 0.7481). The calcified nodule area in Clone 4 was smaller than that of Clones 3 and 16 but was significantly higher in both αMEM(−) and DMEM Cal groups compared to their respective NT groups (*p* = 0.0002 and *p* = 0.0017).

### 3.5. RT-qPCR Verification of Primary Osteoblasts Derived from Calvaria

We performed RT-qPCR to compare the top genes in each medium for POBs derived from calvaria cultured in αMEM(−) and DMEM.

**Figure 5:**
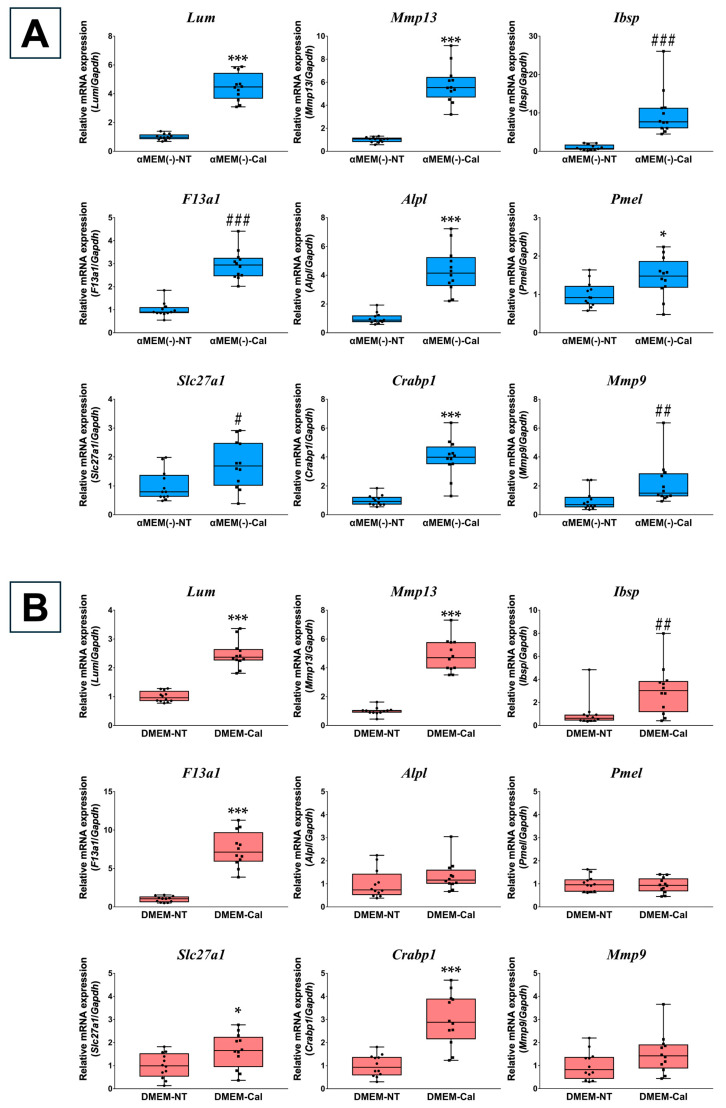

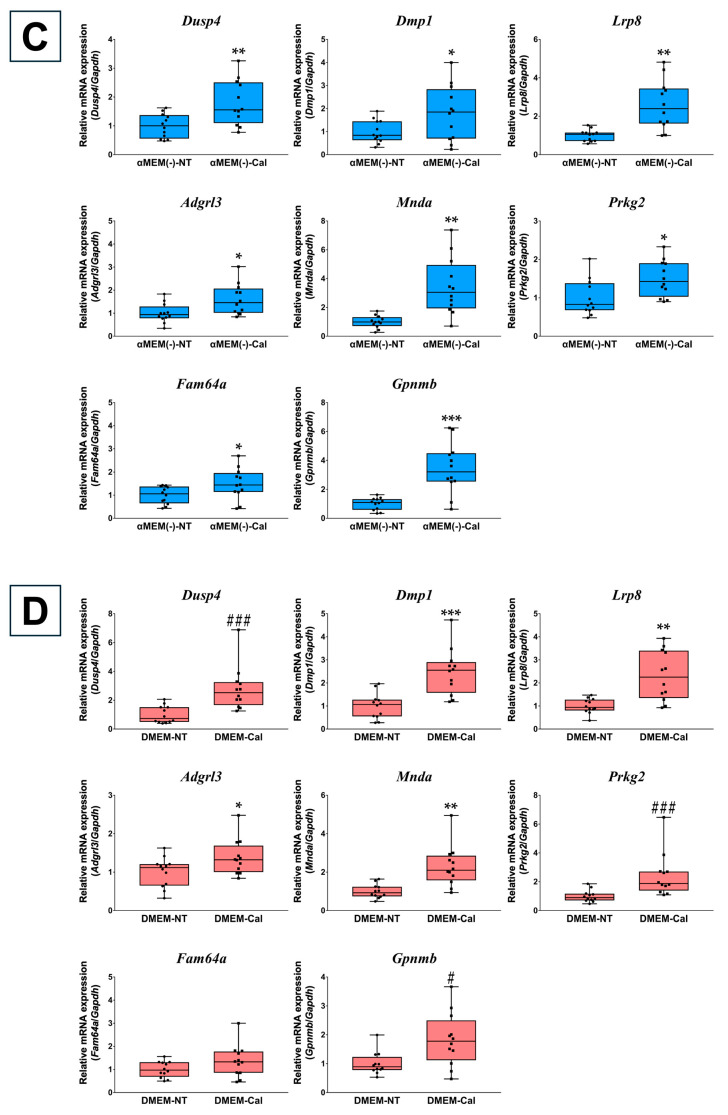

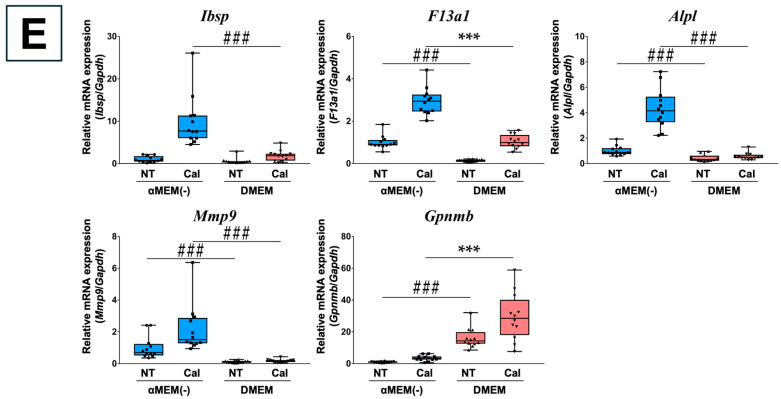
RT-qPCR results of top genes for each medium in primary osteoblasts (POBs) derived from calvaria. (**A**,**B**) RT-qPCR was performed on POBs derived from calvaria cultured in α Minimum Essential Medium without L-ascorbic acid (αMEM(−)) and Dulbecco’s Modified Eagle Medium (DMEM) for the top genes under αMEM(−) culture conditions, based on the microarray results of MC3T3-E1 cells. The expression of target genes was normalized to *Gapdh* expression and calculated by comparing it with the αMEM(−) non-treated group (αMEM(−)-NT) and the DMEM non-treated group (DMEM-NT), respectively. (**C**,**D**) RT-qPCR was performed on POBs cultured in αMEM(−) and DMEM for the top genes under DMEM culture conditions, based on the microarray results of MC3T3-E1 cells. The expression of target genes was normalized to *Gapdh* expression and calculated by comparing it with the αMEM(−)-NT and DMEM-NT groups, respectively. (**E**) Representative genes that showed significant differences in expression between αMEM(−) and DMEM among the top genes from the microarray of MC3T3-E1 cells are shown. Expression of the target genes was normalized to *Gapdh* expression and compared with the αMEM(−)-NT and αMEM(−)-Cal groups, respectively (expression ratios for all groups were referenced to αMEM(−)-NT). Data are presented as box plots showing median values, with whiskers representing the minimum and maximum values. All individual points (*n* = 12) are shown. Statistical significance was evaluated using the Welch’s *t*-test (* *p* < 0.05, ** *p* < 0.01, *** *p* < 0.001) when normality was achieved and the Mann–Whitney U test (# *p* < 0.05, ## *p* < 0.01, ### *p* < 0.001) when normality was not achieved.

For the top genes upregulated in MC3T3-E1 cells cultured in αMEM(−), POBs cultured in αMEM(−) displayed significant expression increases in all genes after calcification stimulation, showing similar trends as in the MC3T3-E1 cells except for *Pmel* (Figure 5A). POBs cultured in DMEM showed significant increases in *Lum* (*p* < 0.0001), *Mmp13* (*p* < 0.0001), *Ibsp* (*p* = 0.0068), *F13a1* (*p* < 0.0001), *Slc27a1* (*p* = 0.0243), and *Crabp1* (*p* < 0.0001)*;* however, *Alpl* was not upregulated (Figure 5B).

Regarding the top genes upregulated in MC3T3-E1 cells cultured in DMEM, significant increases in expression were confirmed in POBs cultured in αMEM(−) for all genes except *Olfr775*, which was not detected (Ct value > 40) (Figure 5C). In POBs cultured in DMEM, significant expression increases were observed for all genes except *Olfr775* and *Fam64a* (Figure 5D). While the expression differences were less pronounced in POBs than in MC3T3-E1 cells, POBs exhibited similar trends of increased expression for DMEM-upregulated genes, regardless of the culture medium.

Comparing relative expression levels between αMEM(−) and DMEM, significant differences were observed for several genes (Figure 5E). *Ibsp*, an osteoblast-related marker, showed a significant difference between the αMEM(−)-Cal and DMEM-Cal groups (*p* < 0.0001). *Alpl* expression levels differed significantly between the αMEM(−)-NT and DMEM-NT groups (*p* = 0.0007), as well as between the αMEM(−)-Cal and DMEM-Cal groups (*p* < 0.0001). Furthermore, *F13a1* and *Mmp9* exhibited significant differences between αMEM(−)-NT and DMEM-NT and between αMEM(−)-Cal and DMEM-Cal (*p* < 0.0001 for all comparisons). *Gpnmb* (osteoactivin) showed markedly higher expression under DMEM conditions, being approximately 16-fold higher in DMEM-NT than in αMEM(−)-NT and 8.5-fold higher in DMEM-Cal than in αMEM(−)-Cal (*p* < 0.0001 for each comparison). These results highlight distinct gene expression patterns between αMEM(−) and DMEM.

### 3.6. Calcification Response of Primary Osteoblasts Derived from Calvaria in Different Media

**Figure 6:**
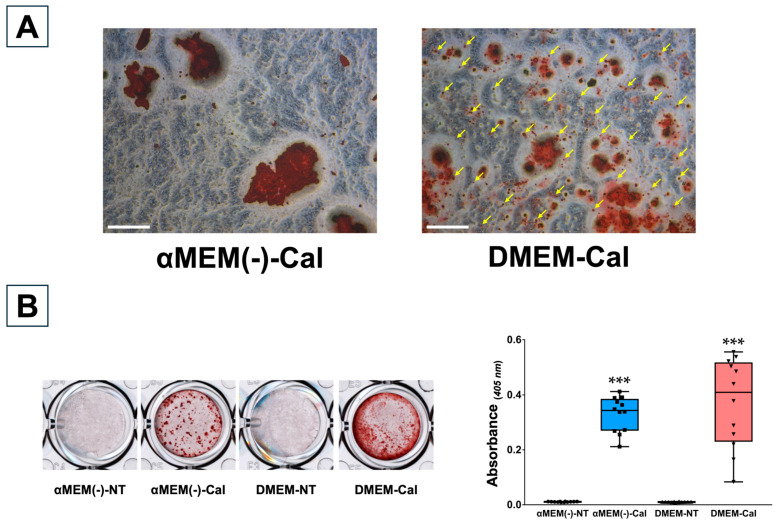
Evaluation of mineralization in primary osteoblasts (POBs) derived from calvaria 35 days after calcification induction in each medium. The mineralization responses of POBs from calvaria cultured in α Minimum Essential Medium without L-ascorbic acid (αMEM(−)) and Dulbecco’s Modified Eagle Medium (DMEM) were compared via Alizarin Red S staining 35 days after calcification induction. After induction, half of the medium was replaced every 3 days. (**A**) Differences in the formation of calcified nodules in αMEM(−) and DMEM. Calcified nodules in each calcification-inducing medium were observed under an optical microscope. Yellow arrowheads indicate widespread calcified nodule granules. Scale bar: 500 µm. Calcification-inducing group: Cal. (**B**) On the left, representative images of Alizarin Red S staining of POBs under each culture condition. On the right, the absorbance at 405 nm of a solution of mineral nodules dissolved in 5% formic acid by stirring for 10 min is shown. Data are presented as box plots showing median values, with whiskers representing the minimum and maximum values. All individual points (*n* = 12) are shown. Statistical significance was evaluated using the Welch’s *t*-test (*** *p* < 0.001). Non-treated group: NT, calcification induction group: Cal.

POBs cultured in αMEM(−) and DMEM were stained with Alizarin Red S on the 35th day after calcification-inducing stimulation to assess the calcification response. Calcified nodules were not observed in any NT group, whereas prominent nodules were formed in the Cal group. Under optical microscopy, POBs cultured in αMEM(−) exhibited localized island-like nodules, whereas POBs cultured in DMEM showed small, widely distributed stained granules, indicating distinct calcification patterns between the media (Figure 6A). Representative calcification images and the quantification of calcified nodules dissolved in formic acid are shown in Figure 6B. Quantitative analysis revealed significantly higher calcification in the Cal group compared to the NT group for both media (*p* < 0.0001 for each comparison).

## 4. Discussion

In the MC3T3-E1 experiment, based on our previous report [12], we selected a cell concentration by which the cell proliferation ability due to differences in culture medium would not significantly affect the osteoblast differentiation state. αMEM and DMEM are frequently used for culturing MC3T3-E1, but the main differences between them are the concentrations of amino acids, nucleosides, and vitamins. Orriss et al. have suggested that proline, which is necessary for collagen synthesis and is only found in αMEM, may cause differences in the bone formation activity of POBs [18]. Therefore, we expected that the differences in these medium components would also affect gene expression in the preosteoblast cell line MC3T3-E1 cells. When MC3T3-E1 cells were cultured in αMEM(−) or DMEM and subjected to mineralization stimulation, significant differences in gene expression patterns were observed. Notably, microarray results after mineralization stimulation in DMEM did not show increases in established osteoblast differentiation markers, such as *Runx2* and *Alpl* [19]. Taken together with our previous data, we demonstrated that MC3T3-E1 cells underwent mineralization through the stimulation of the calcification, regardless of the medium type; these findings suggest that osteoblast differentiation markers are not necessarily direct indicators of mineralization. Many studies employ osteoblast differentiation markers in the in vitro evaluation of biomaterials and implants; however, our results indicate that such markers may not adequately assess mineralization potential [20,21]. Instead, the top 10 genes with the most pronounced changes in response to each medium in this study are likely osteoblast response genes activated by L-ascorbic acid stimulation. Supporting this hypothesis, the gene expression analysis after mineralization stimulation of POBs cultured in L-ascorbic acid-free αMEM and DMEM revealed increases in most of the top genes identified in MC3T3-E1 cells, with the exception of *Alpl*. Interestingly, *Alpl* expression did not increase following mineralization stimulation in POBs cultured in DMEM, a pattern consistent with MC3T3-E1 cells cultured in DMEM. *Alpl,* which plays a key role in phosphate supply during bone formation, is considered essential for mineralization [22]. Nevertheless, studies on *Alpl* knockout mice indicate that while bone formation is abnormal, mineralization is not entirely absent [23,24]. These findings suggest the existence of *Alpl*-independent pathways of bone formation in vivo. The *Alpl* unchanged event observed in MC3T3-E1 cells cultured in DMEM, as well as the mineralization of Clone 16 and POBs cultured in DMEM, may reflect these alternative pathways. The specific role and the extent of this *Alpl*-independent calcification process in physiological bone formation remain unclear and warrant further investigation. However, we propose that the use of osteoblasts cultured in DMEM may enable the study of *Alpl*-independent calcification mechanisms.

POBs have gained traction as an in vitro model closer to in vivo conditions for evaluating biomaterials and implants [25,26]. Several studies reference Bakker et al.’s protocol in “Bone Research Protocols” for extracting POBs [27,28]. This protocol involves culturing POBs in DMEM supplemented with L-ascorbic acid from the point of extraction. The gene expression profiles observed in this study were derived from POBs cultured under L-ascorbic acid-free conditions and reflect the state of L-ascorbic acid-responsive genes similar to that observed in MC3T3-E1 cells. When evaluating implants using POBs harvested via Bakker et al.’s method, it should be assumed that mineralization stimulation has already occurred. Through this study, we have also confirmed that the calcification stimulation of POBs harvested in DMEM containing L-ascorbic acid yielded no remarkable changes in the expression of numerous genes, including osteogenesis-related genes. This outcome is consistent with previous findings showing that culturing osteoblastic cells in a L-ascorbic acid-containing medium induces the expression of genes known as differentiation-related genes even without additional stimulation [12]. Therefore, the duration of proliferation culture before evaluating biomaterials or implants may significantly influence the results.

The suitability of MC3T3-E1 cells versus POBs for biomaterial evaluation was assessed by comparing gene expression responses to L-ascorbic acid stimulation under varying culture medium conditions. In MC3T3-E1 cells, gene upregulation due to L-ascorbic acid stimulation varied markedly depending on the culture medium. These osteoblastic cells were derived from populations already exposed to L-ascorbic acid during their establishment [2], and their heterogeneity has been well documented [7,9]. This inherent variability likely contributes to the existence of subpopulations, such as Clone 1, that do not respond to additional mineralization stimuli. Conversely, clone experiments revealed that some MC3T3-E1 cells could respond to L-ascorbic acid stimulation, particularly when cultured in a L-ascorbic acid-free medium during proliferation. However, most calcifying clones preferentially responded to specific media, suggesting that the reversibility of calcification stimuli is influenced by the base medium. For instance, in POBs cultured in a L-ascorbic acid-free medium prior to mineralization stimulation, genes related to bone formation, such as *Alpl* and *Gpnmb,* exhibited differential expression. The cultivation of POBs in a L-ascorbic acid-free medium requires high cell densities, often necessitating the use of numerous animals, making it impractical for the large-scale screening of biomaterials. In contrast, MC3T3-E1 cells are easily accessible and demonstrate reset gene expression recognized to be related to osteoblast differentiation when cultured in a L-ascorbic acid-free medium. This makes them more practical for biomaterial screening.

Gene expression analysis in animal models suggests that MC3T3-E1 cells cultured in αMEM-based formulations respond to mineralization stimuli in ways that reflect physiological processes such as bone development and fracture healing [13,29]. Conversely, genes such as *Dmp1* and *Gpnmb,* whose expression changed in response to mineralization stimuli in MC3T3-E1 cells cultured in DMEM, have been implicated in bone formation, though their roles in vivo remain poorly understood [30,31]. Interestingly, compensatory bone formation in hypophosphatasia patients is thought to occur independently of *Alpl* [32], indicating that MC3T3-E1 cells cultured in DMEM might be particularly useful for evaluating biomaterials and therapeutics for such conditions in the field of orthopedics. Further studies are warranted, including histological evaluations focusing on genes extracted with DMEM in animal models of various bone formation situations, as well as in vitro investigations aimed at identifying genes truly related to osteoblast differentiation and calcification to expand this understanding.

One limitation of this study is that it only investigated the difference in status at one time point, 3 days after the calcification stimulus, and so the changes over time were unclear. In addition, no detailed investigation was conducted into the differences in products such as cell matrix that are brought about by variations in gene expression. As such, the direct relationship with calcification is partially unclear. Future research is needed to clarify these points.

## 5. Conclusions

Comprehensive gene expression analyses in MC3T3-E1 cells cultured in different media revealed distinct top-ranking genes in each case, underscoring the impact of medium conditions on in vitro bone formation processes. The in vitro development evaluation of novel biomaterials for bone disease treatment requires the careful consideration of culture medium conditions, as these significantly influence gene expression during mineralization. In addition, while most of the top genes in MC3T3-E1 cells showed upregulation in POBs cultured in both media, they appear to be responsive to L-ascorbic acid stimulation rather than direct markers of calcification. Variations in *Alpl* expression, a known osteoblast differentiation marker, and differences in mineralized nodule formation were also observed. It is a very interesting result that there can be an osteoblast state with mineralization ability without increasing *Alpl*, a known differentiation indicator, and this may correspond to each process of bone formation in the body. In other words, the distinct gene expression profiles in MC3T3-E1 cells may represent multiple bone formation pathways, both in vitro and in vivo. Future investigations on these genes could pave the way for more accurate screening methods for implant evaluation in clinical orthopedics, addressing a critical need in clinical practice.

## Figures and Tables

**Table 1:** Primer information.

Gene Symbol	Gene Name	Takara Bio Primer_Set ID
*Gapdh*	Glyceraldehyde-3-phosphate dehydrogenase	MA050371
*Lum*	Lumican	MA206078
*Mmp13*	Matrix metallopeptidase 13	MA198906
*Ibsp*	Integrin binding sialoprotein	MA220893
*F13a1*	Coagulation factor XIII, A1 subunit	MA217968
*Alpl*	Alkaline phosphatase, liver/bone/kidney	MA127137
*Pmel*	Premelanosome protein	MA232167
*Slc27a1*	Solute carrier family 27 (fatty acid transporter), member 1	MA201988
*Crabp1*	Cellular retinoic acid binding protein I	MA211028
*Mmp9*	Matrix metallopeptidase 9	MA031311
*Dusp4*	Dual specificity phosphatase 4	MA089467
*Dmp1*	Dentin matrix protein 1	MA155586
*Lrp8*	Low-density lipoprotein receptor-related protein 8, apolipoprotein E receptor	MA203890
*Adgrl3*	Adhesion G protein-coupled receptor L3	MA237640
*Mnda*	Interferon-activated gene 211	MA197419
*Olfr775*	Olfactory receptor family 6 subfamily C member 205	MA231969
*Prkg2*	Protein kinase, cGMP-dependent, type II	MA220730
*Fam64a*	PICALM interacting mitotic regulator	MA235428
*Gpnmb*	Glycoprotein (transmembrane) nmb	MA212827

**Table 2:** Top 10 genes significantly upregulated 3 days after calcification stimulation in MC3T3-E1 cells cultured in α Minimum Essential Medium without L-ascorbic acid (αMEM(−)) and Dulbecco’s Modified Eagle Medium (DMEM), as identified by microarray analysis.

**αMEM(** **−** **) Top 10 Genes**					
**Gene** **Symbol**	**Gene ID**	**mRNA** **Accession**	**Name**	**Fold Change**	
				**αMEM**(−)	**DMEM**
*Lum*	17022	NM_008524	Lumican	8.62	−1.32
*Mmp13*	17386	NM_008607	Matrix metallopeptidase 13	7.34	1.43
*Ibsp*	15891	NM_008318	Integrin binding sialoprotein	6.17	−1.25
*F13a1*	74145	NM_001166391	Coagulation factor XIII, A1 subunit	5.38	1.38
*Alpl*	11647	NM_001287172	Alkaline phosphatase, liver/bone/kidney	4.93	−1.17
*Pmel*	20431	NM_021882	Premelanosome protein	4.61	1.49
*Slc27a1*	26457	NM_011977	Solute carrier family 27 (fatty acid transporter), member 1	4.06	1.54
*Crabp1*	12903	NM_001284507	Cellular retinoic acid binding protein I	3.92	1.95
*Mmp9*	17395	NM_013599	Matrix metallopeptidase 9	3.74	−1.41
*Gm11099*	-	ENSMUST 00000112589	Predicted gene 11099	3.57	−1.47
**DMEM Top 10 Genes**					
**Gene** **Symbol**	**Gene ID**	**mRNA** **Accession**	**Name**	**Fold Change**	
				**αMEM**(−)	**DMEM**
*Dusp4*	319520	NM_176933	Dual specificity phosphatase 4	−1.49	10.67
*Dmp1*	13406	NM_016779	Dentin matrix protein 1	2.10	9.35
*Lrp8*	16975	NM_001080926	Low-density lipoprotein receptor-related protein 8, apolipoprotein E receptor	−1.07	6.13
*Adgrl3*	319387	NM_198702	Adhesion G protein-coupled receptor L3	−7.91	5.78
*Mnda*	381308	NM_001033450	Interferon-activated gene 211	1.07	5.59
*Olfr775*	258538	NM_146545	Olfactory receptor family 6 subfamily C member 205	1.10	5.00
*Prkg2*	19092	NM_008926	Protein kinase, cGMP-dependent, type II	1.43	5.00
*Fam64a*	109212	NM_144526	PICALM interacting mitotic regulator	−1.33	4.97
*Gm13212*	433801	NM_001205101	Predicted gene 13212	1.50	4.81
*Gpnmb*	93695	NM_053110	Glycoprotein (transmembrane) nmb	2.09	4.69

## Data Availability

The original contributions presented in this study are included in the article. Further inquiries can be directed to the corresponding author.

## Abbreviations

The following abbreviations are used in this manuscript:

αMEM	α Minimum Essential Medium
αMEM(−)	α Minimum Essential Medium without L-ascorbic acid
DMEM	Dulbecco’s Modified Eagle Medium
DPBS	Dulbecco’s Phosphate-Buffered Saline
FBS	Fetal bovine serum
*Gapdh*	Glyceraldehyde-3-phosphate dehydrogenase
POB	Primary osteoblast
POBs	Primary osteoblasts
